# P-1377. Utility of Xpert MTB/XDR Assay in Patients with Rifampicin Indeterminate Results on Xpert MTB/Rif Ultra with Trace Load

**DOI:** 10.1093/ofid/ofaf695.1564

**Published:** 2026-01-11

**Authors:** Dhruv Gandhi, Ira Shah

**Affiliations:** Bai Jerbai Wadia Hospital for Children, Mumbai, India, West Monroe, LA; Bai Jerbai Wadia Hospital for Children, Mumbai, India, West Monroe, LA

## Abstract

**Background:**

Rapid nucleic acid amplification tests (NAAT), such as Xpert MTB/Rif, Xpert Ultra, and Xpert MTB/XDR, are critical for diagnosing pediatric tuberculosis (TB) and detecting drug resistance. However, rifampicin indeterminate (RI) results, particularly in samples with trace Mycobacterium tuberculosis (MTB) load, pose a clinical challenge. Current guidelines do not recommend performing Xpert MTB/XDR in patients with prior trace calls on Xpert Ultra. The aim of this study is to analyze the drug-resistance results on Xpert MTB/Rif or Ultra and Xpert MTB/XDR, particularly the results of Xpert MTB/XDR in those patients with prior RI results on Xpert MTB/Rif or Ultra and in those with trace calls on Xpert Ultra.

**Table 1:**
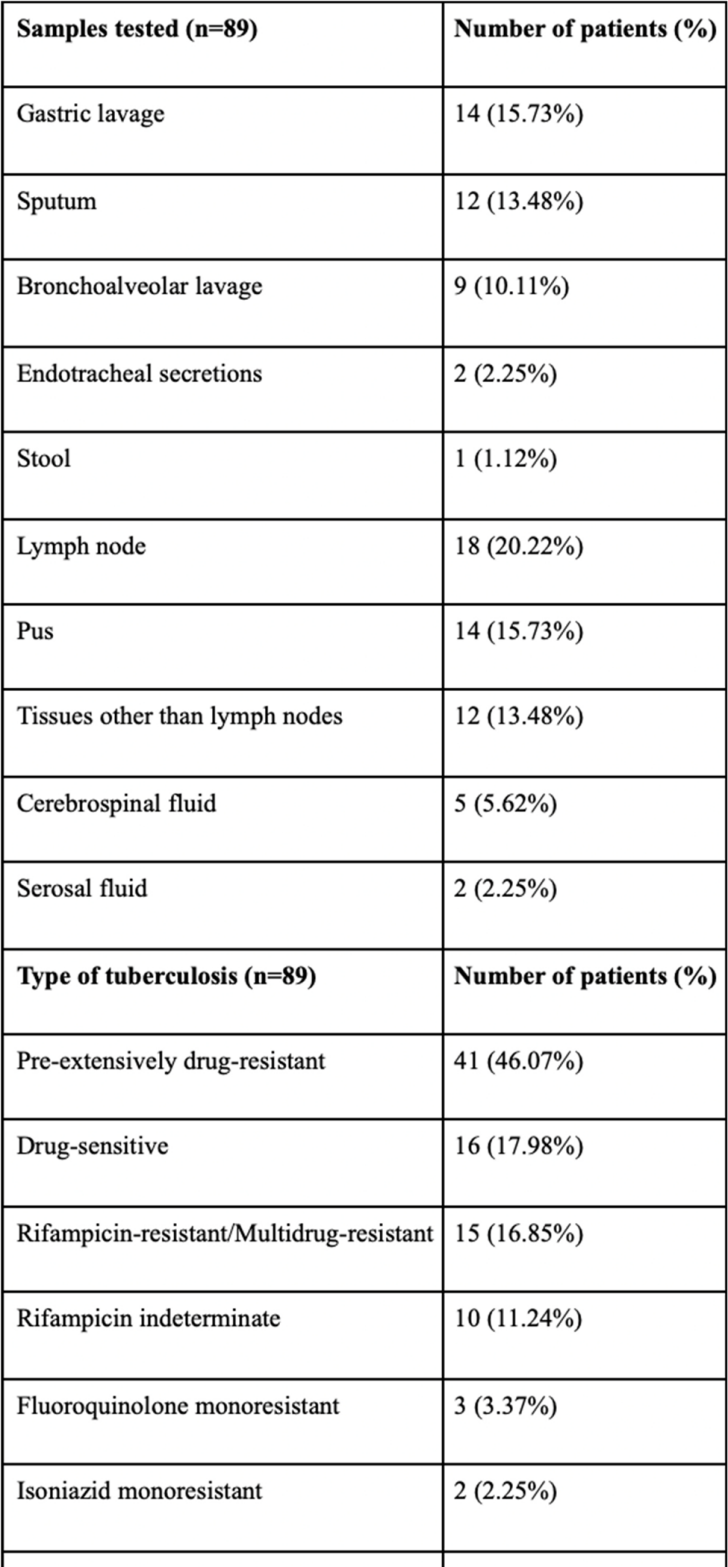
Samples tested and the type of tuberculosis in the patients based on the results of Xpert MTB/Rif or Ultra and Xpert MTB/XDR assays

**Table 2:**
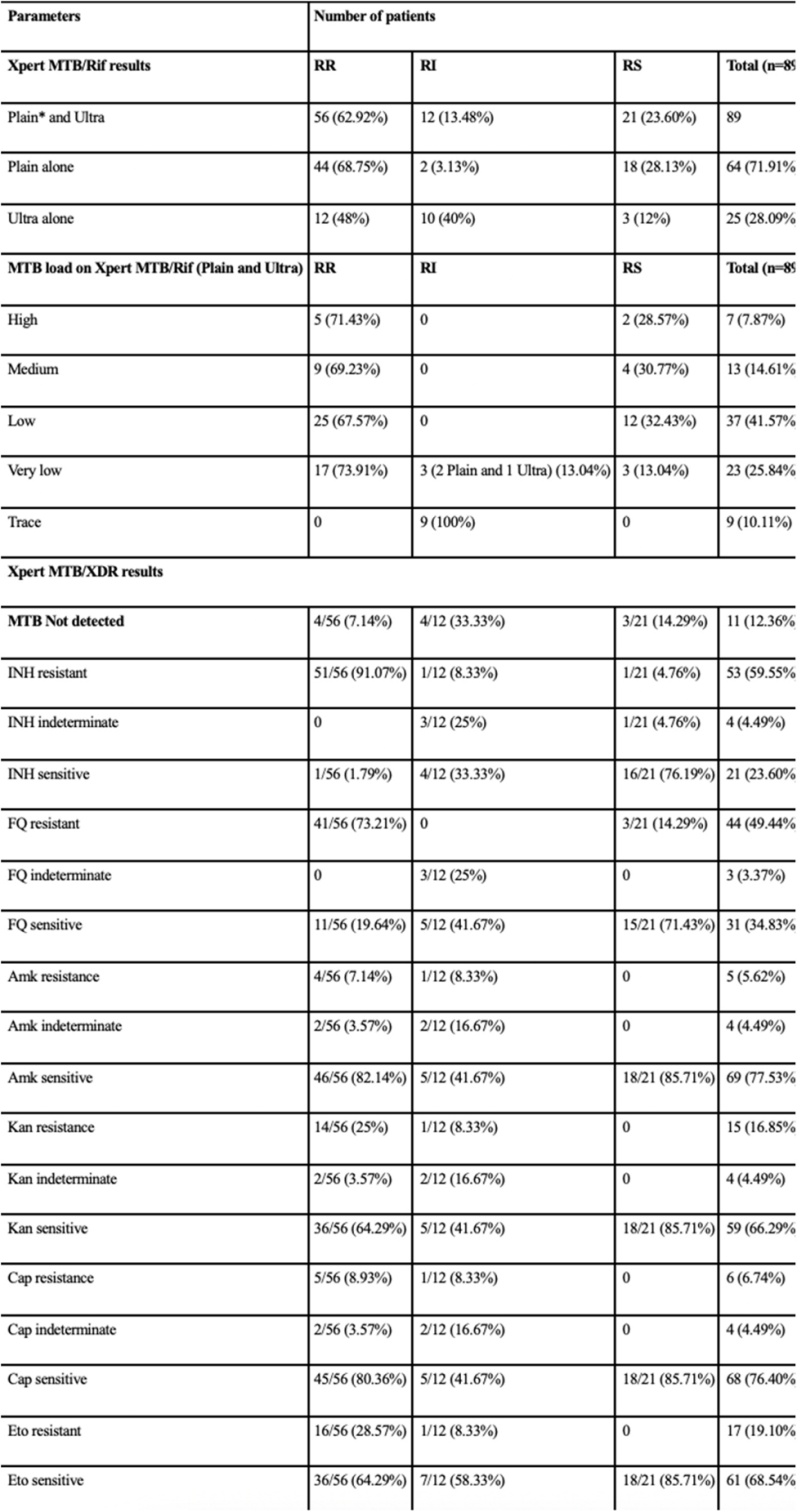
Results of Xpert MTB/Rif or Ultra assay (rifampicin resistance, MTB load) and Xpert MTB/XDR panel Note: MTB- Mycobacterium tuberculosis, RR- Rifampicin resistant, RI- Rifampicin indeterminate, RS- Rifampicin sensitive, *Plain- refers to Xpert MTB/Rif, INH- Isoniazid, FQ- Fluoroquinolone, Amk- Amikacin, Kan- Kanamycin, Cap- Capreomycin, Eto- Ethionamide.

**Methods:**

A retrospective study was conducted at a pediatric TB clinic in Mumbai, India, including 89 children less than 18 years of age, diagnosed with TB, between October 2020 and August 2024. All patients were positive on Xpert MTB/Rif or Ultra and underwent Xpert MTB/XDR testing. Data on rifampicin resistance, MTB load, and resistance to other drugs were analyzed.

**Results:**

Fifty-six(62.92%) patients were rifampicin resistant (RR), 12(13.5%) were RI, and 21(23.6%) were rifampicin sensitive(RS). Xpert MTB/XDR did not detect Mycobacetrium tuberculosis(MTB) in 4(7.14%) patients with RR, 4(33.3%) patients with RI, and 3(14.29%) patients with RS results. Of 12 RI results on Xpert MTB/Rif or Ultra, 3(25%) had very low MTB load and 9(75%) had trace MTB load. Of the 9 samples with trace results, Xpert MTB/XDR detected MTB in 5(55.56%) of which 2(40%) had pan-indeterminate drug-resistance results on Xpert MTB/XDR, 2(40%) were pan-sensitive on Xpert MTB/XDR, and 1(20%) was sensitive to isoniazid and fluoroquinolones, but resistant to second-line injectable drugs and ethionamide. Thus, 3 out of 9(33.33%) patients with trace calls had actionable results on Xpert MTB/XDR.

**Conclusion:**

Both, RI results on Xpert MTB/Rif or Ultra and non-detection of MTB on Xpert MTB/XDR, are associated with lower MTB-loads on Xpert MTB/Rif or Ultra. Xpert MTB/XDR is useful in clinical decision-making in one-third of the patients with prior RI trace calls on Xpert Ultra and may be useful in devising an antitubercular therapy regimen when a second sample may not be easily available for testing.

**Disclosures:**

All Authors: No reported disclosures

